# Naturally Occurring Angiotensin Peptides Enhance the SARS-CoV-2 Spike Protein Binding to Its Receptors

**DOI:** 10.3390/ijms26136067

**Published:** 2025-06-24

**Authors:** Katelin X. Oliveira, Fariha E. Bablu, Emily S. Gonzales, Taisuke Izumi, Yuichiro J. Suzuki

**Affiliations:** 1 Department of Pharmacology and Physiology, Georgetown University Medical Center, Washington, DC 20057, USA; kxo2@georgetown.edu (K.X.O.); fb522@georgetown.edu (F.E.B.); esg67@georgetown.edu (E.S.G.); 2 Department of Biology, College of Arts & Sciences, American University, Washington, DC 20016, USA; izumi@american.edu; 3 District of Columbia Center for AIDS Research, Washington, DC 20052, USA

**Keywords:** angiotensin, AXL, COVID-19, peptide, SARS-CoV-2, spike protein

## Abstract

Severe Acute Respiratory Syndrome Coronavirus 2 (SARS-CoV-2), the virus responsible for Coronavirus Disease 2019 (COVID-19), utilizes its spike protein to infect host cells. In addition to angiotensin-converting enzyme 2 (ACE2) and neuropilin-1 (NRP1), AXL acts as a spike protein receptor and mediates infection, especially in respiratory cells with low ACE2 expression. Angiotensin II (1–8) can be cleaved into shorter peptides within the biological system. Antibody-based binding assays showed that angiotensin II causes a two-fold increase in the binding between the spike protein and AXL, but not ACE2 or NRP1. While a longer peptide, angiotensin I (1–10), did not affect the spike–AXL binding, shorter lengths of angiotensin peptides exhibited enhancing effects. The *C*-terminal deletions of angiotensin II to angiotensin (1–7) or angiotensin (1–6) resulted in peptides with enhanced activity toward spike–AXL binding with a similar capacity as angiotensin II. In contrast, the *N*-terminal deletions of angiotensin II to angiotensin III (2–8) or angiotensin IV (3–8) as well as the *N*-terminal deletions of angiotensin (1–7) to angiotensin (2–7) or angiotensin (5–7) produced peptides with a more potent ability to enhance spike–AXL binding (2.7-fold increase with angiotensin IV). When valine was substituted for tyrosine at position 4 in angiotensin II or when tyrosine at position 4 was phosphorylated, spike–AXL binding was increased, suggesting that modifications to tyrosine trigger enhancement. Angiotensin IV also enhances spike protein binding to ACE2 and NRP1. Thus, angiotensin peptides may contribute to COVID-19 pathogenesis by enhancing spike protein binding and thus serve as therapeutic targets.

## 1. Introduction

Severe Acute Respiratory Syndrome Coronavirus 2 (SARS-CoV-2) is a positive-sense, single-stranded RNA virus of the genus *Betacoronavirus* responsible for causing the Coronavirus Disease 2019 (COVID-19) pandemic [1,2]. This virus consists of several structural proteins: nucleocapsid protein, membrane protein, envelope protein, and spike protein [1]. The spike protein is the viral membrane fusion protein of SARS-CoV-2 that recognizes and fuses with host cell receptors [1,2]. The spike protein comprises two subunits: the S1 subunit and the S2 subunit [1,2]. The S1 subunit contains the receptor-binding domain, which directly interacts with the major host cell receptor angiotensin-converting enzyme 2 (ACE2) [2]. The S2 subunit anchors the spike protein to the viral membrane [1] and mediates membrane fusion [2]. Following receptor binding, the spike protein is cleaved by transmembrane protease serine 2, which activates membrane fusion and allows the release of the viral ribonucleoprotein complex into the host cell [2].

**Figure 1:**
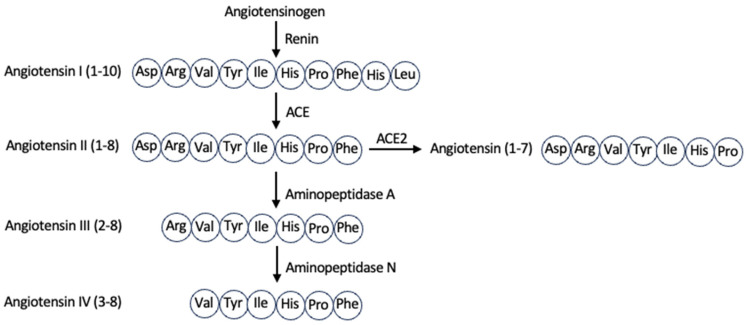
Structures of the angiotensin peptides. This figure illustrates the structures of the naturally occurring angiotensin peptides relevant to the experiments performed in this study. It also outlines how angiotensin I (1–10), angiotensin II (1–8), angiotensin (1–7), angiotensin III (2–8), and angiotensin IV (3–8) are physiologically synthesized in the biological system.

Angiotensin peptides are key components of the renin–angiotensin system (RAS), a regulatory pathway that is critical for maintaining cardiovascular and renal functions [3,4]. In the classical RAS pathway, angiotensinogen, a prohormone synthesized in the liver, is cleaved by renin in the kidney to produce angiotensin I (1–10) [3,4,5]. Angiotensin I (1–10) consists of the following ten amino acids: Asp, Arg, Val, Tyr, Ile, His, Pro, Phe, His, and Leu [4,6]. Angiotensin-converting enzyme (ACE) cleaves angiotensin I (1–10) between His9 and Leu10, yielding angiotensin II (1–8) [3,4,5,6], which includes Asp, Arg, Val, Tyr, Ile, His, Pro, and Phe [3,4,6], as shown in Figure 1. Angiotensin II (1–8) primarily exerts its biological effects by binding to the type 1 angiotensin II receptor (AT1R), a G protein-coupled receptor (GPCR). This interaction results in smooth muscle contraction, increased production of aldosterone and antidiuretic hormone (ADH), enhanced sympathetic nervous system tone, elevated blood pressure, cardiac hypertrophy and fibrosis, decreased nitric oxide (NO) production, parasympathetic nervous system tone, baroreflex sensitivity, and natriuresis [4,6,7]. Angiotensin II (1–8) also binds to another GPCR, the type 2 angiotensin II receptor (AT2R), which counteracts many of the effects mediated through AT1R [3,5,7]. The activation of AT2R is associated with vasodilation, anti-fibrotic, anti-inflammatory, and anti-proliferative effects [3,4,5,7]. Additionally, angiotensin II (1–8) stimulates the generation of reactive oxygen species and various cellular processes, including proliferation, differentiation, apoptosis, and inflammation [5,7].

Angiotensin II (1–8) can also be cleaved by aminopeptidase A at Asp1, producing angiotensin III (2–8) [3,4,5,7], as shown in Figure 1. Angiotensin III (2–8) acts through AT1R and AT2R [4,5], increasing blood pressure and ADH release, although with lower potency compared to angiotensin II (1–8) [4,5,7]. Subsequent cleavage by aminopeptidase N removes Arg2 from angiotensin III (2–8) to form angiotensin IV (3–8) [3,4,5]. Angiotensin IV (3–8) binds to the type 4 angiotensin II receptor [3,4,5], which is expressed in the brain, kidney, heart, and blood vessels [5]. This interaction enhances natriuresis, stimulates NO production, and promotes cardioprotective effects, such as reduced vasoconstriction [4]. In addition to contributing to blood flow regulation [3,5], angiotensin IV (3–8) has also been implicated in cognitive functions such as learning [3,8], memory [3,4,8], and neuronal development [3,8]. The phenylalanine at position 8 in angiotensin II (1–8) can also be cleaved off by ACE2 to produce angiotensin (1–7) (Figure 1), a vasodilator [3,4] that binds to the Mas GPCR [3,6].

In addition to its role in hemodynamic regulation and angiotensin II (1–8) metabolism, ACE2 is a well-established receptor for SARS-CoV-2 entry into host cells [1,2,9,10], establishing a link between COVID-19 and RAS. SARS-CoV-2 infection of human airway epithelial cells correlates with the presence of well-differentiated cells that express high levels of ACE2 [11]. However, ACE2 expression is relatively low in respiratory tissues, especially in the lungs and bronchi, suggesting that other receptors may facilitate viral entry [12,13,14].

One candidate receptor for SARS-CoV-2 is AXL [14], a receptor tyrosine kinase belonging to the TAM family [14,15,16,17,18]. AXL mediates various cellular signaling pathways and is involved in biological processes such as proliferation, migration, differentiation, and apoptosis [15,16]. Initially identified in patients with chronic myeloid leukemia [15,16,17,18,19], AXL is predominantly studied in cancer research. AXL expression has been detected in vascular and immune cells [15], embryonic cells [16], tumor cells, bone marrow stoma [17], fibroblasts, myeloid progenitor cells, neural tissue, cardiac muscle, and skeletal muscle [18]. Notably, AXL is highly expressed in respiratory cells, unlike ACE2 [14], suggesting its importance in infections by respiratory viruses such as SARS-CoV-2. Furthermore, studies suggest that AXL expression may be upregulated by GPCR agonists, such as angiotensin II (1–8) and thrombin [19]. AXL was found to interact with the *N*-terminal domain of the spike protein and participate in the SARS-CoV-2 infection of the human respiratory system [14]. In addition to serving as a potential SARS-CoV-2 receptor, AXL has been proposed as a molecular marker for COVID-19 progression [20].

The present study reports that angiotensin II (1–8) and other naturally occurring angiotensin peptides enhance the binding of the SARS-CoV-2 spike protein to AXL.

## 2. Results

### 2.1. Effects of Angiotensin II (1–8) on Spike Protein Binding to Host Cell Receptors

**Figure 2:**
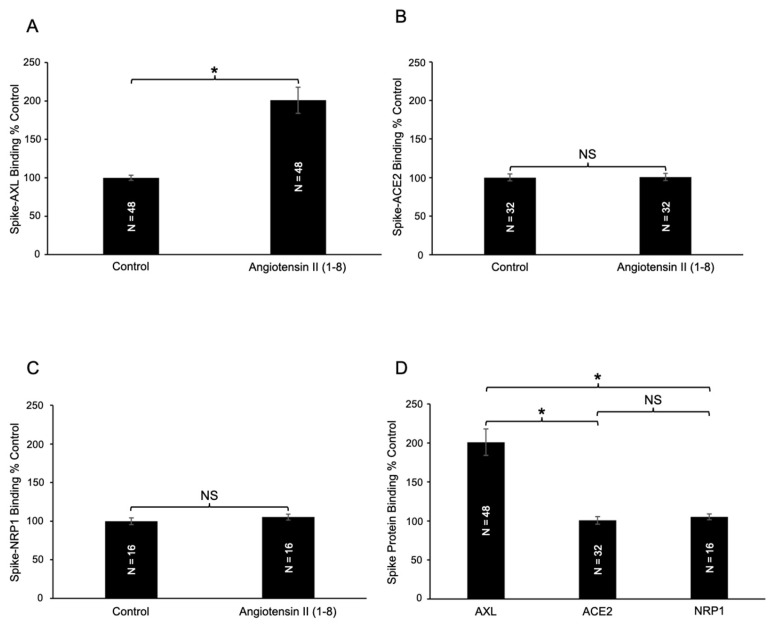
Effects of angiotensin II (1–8) on spike protein binding. (**A**) The addition of angiotensin II (1–8) significantly enhances spike–AXL binding. In contrast, angiotensin II (1–8) did not significantly alter (**B**) spike–ACE2 binding or (**C**) spike–NRP1 binding, respectively. (**D**) The effect of angiotensin II (1–8) on spike protein binding across three different receptors. Angiotensin II (1–8) selectively enhanced spike–AXL binding and did not influence spike–ACE2 binding or spike–NRP1 binding. The bar graphs represent means ± SEM. An asterisk (*) denotes that the values are significantly different from each other at *p* < 0.05, and “NS” indicates “not significant”.

The effects of angiotensin II (1–8) on the spike protein binding to a host cell receptor AXL were examined using an antibody-based colorimetric microplate assay. In this assay, AXL protein is coated at the bottom of the wells of a 96-well microplate. The spike protein is then added to the wells, washed, and the binding of the spike protein to AXL is monitored using the primary antibody that detects the spike protein and the secondary antibody covalently bound to HRP, which produces color in the presence of the TMB substrate. Angiotensin II was added before the addition of the spike protein. Our results using this assay revealed that angiotensin II (1–8) significantly enhanced spike protein–AXL binding (Figure 2A). A two-fold increase was observed.

We also performed spike protein binding assays to test the effects of angiotensin II (1–8) on interactions with other known spike protein receptors, ACE2 and neuropilin-1 (NRP1) [1]. In contrast to AXL, no significant changes were observed for the spike protein–ACE2 binding (Figure 2B) or spike protein–NRP1 binding (Figure 2C). Thus, the enhancing effect of angiotensin II (1–8) is specific to spike–AXL binding. Figure 2D shows that the differences between the effect of angiotensin II (1–8) on spike–AXL binding and spike–ACE2 binding, as well as the difference between spike–AXL binding and spike–NRP1 binding, were significantly different.

**Figure 3:**
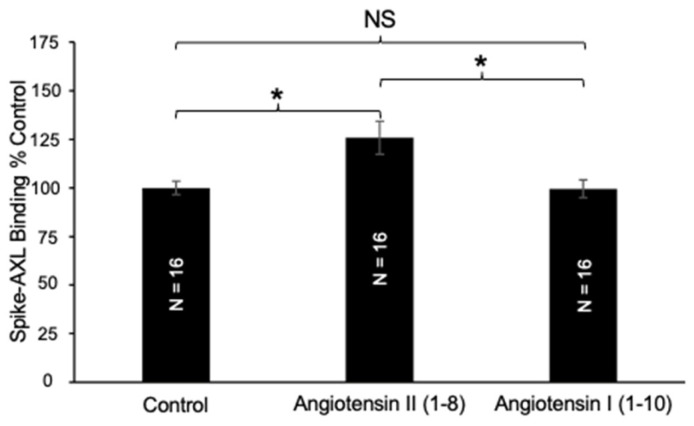
Effects of angiotensin I (1–10) on spike–AXL binding. Only angiotensin II (1–8) increases spike–AXL binding, while angiotensin I (1–10) does not. The bar graphs represent means ± SEM. An asterisk (*) denotes that the values are significantly different from each other at *p* < 0.05, and “NS” indicates “not significant”.

We also found that a longer angiotensin peptide, angiotensin I (1–10), the precursor of angiotensin II (1–8) in the ACE reaction, did not affect the spike–AXL binding (Figure 3). Therefore, these experiments illustrated that angiotensin II (1–8) has an enhancing effect on spike–AXL binding, while a longer angiotensin peptide, angiotensin I (1–10), did not influence spike–AXL binding.

### 2.2. Effects of C-Terminal Deletion of Angiotensin II (1–8) on Spike–AXL Binding

**Figure 4:**
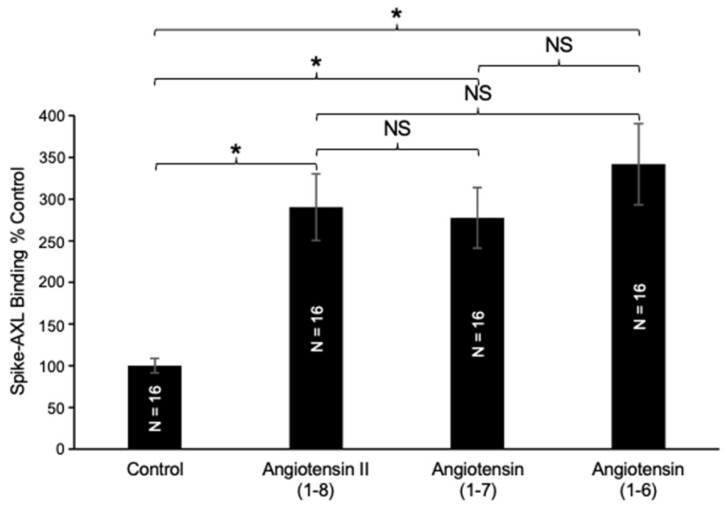
Effects of angiotensin (1–7) and angiotensin (1–6) on spike–AXL binding. Angiotensin II (1–8), angiotensin (1–7), and angiotensin (1–6) all significantly enhance spike–AXL binding. However, when compared to each other, the enhancing effects of angiotensin II (1–8), angiotensin (1–7), and angiotensin (1–6) are not significantly different from one another. The bar graphs represent means ± SEM. An asterisk (*) denotes that the values are significantly different from each other at *p* < 0.05, and “NS” indicates “not significant”.

To investigate structure–function relationships, we evaluated the effects of truncated forms of angiotensin II (1–8) on spike–AXL binding. In the biological systems, the *C*-terminal phenylalanine of angiotensin II (1–8) is cleaved by ACE2 to produce angiotensin (1–7) (Figure 1). Our results in Figure 4 showed that angiotensin (1–7) also enhanced the spike–AXL binding and that its effect was similar to angiotensin II (1–8), suggesting that the *C*-terminal deletion of phenylalanine at position 8 from angiotensin II (1–8) did not modify the enhancing effect on spike–AXL binding. A further *C*-terminal deletion to produce angiotensin (1–6) did not alter the enhancing effect either (Figure 4).

### 2.3. Effects of N-Terminal Deletion of Angiotensin II (1–8) on Spike–AXL Binding

**Figure 5:**
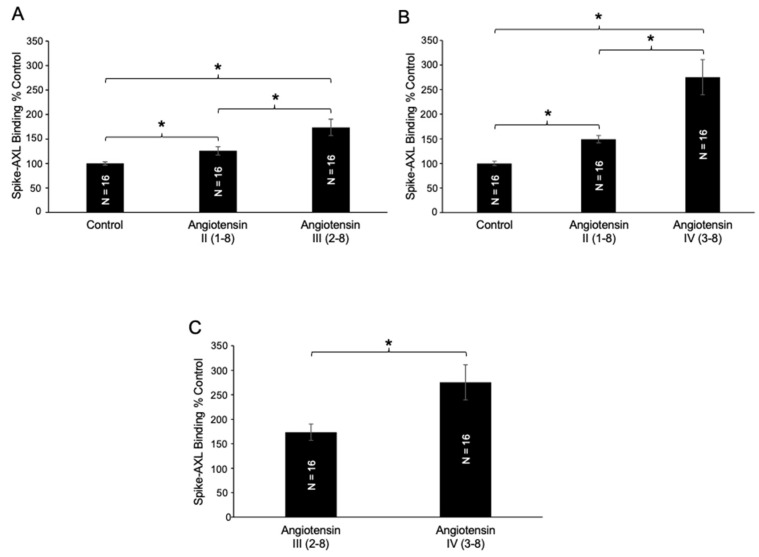
Effects of angiotensin III (2–8) and angiotensin IV (3–8) on spike–AXL Binding. (**A**) Both angiotensin II (1–8) and angiotensin III (2–8) enhance spike–AXL binding, with angiotensin III (2–8) enhancing spike–AXL binding more potently than angiotensin II (1–8). (**B**) Both angiotensin II (1–8) and angiotensin IV (3–8) significantly increase spike–AXL binding, with angiotensin IV (3–8) enhancing spike–AXL binding more potently than angiotensin II (1–8). (**C**) Angiotensin IV (3–8) enhances spike–AXL binding more potently than angiotensin III (2–8). The bar graphs represent means ± SEM. An asterisk (*) denotes that the values are significantly different from each other at *p* < 0.05.

The *N*-terminal deletion of an amino acid from angiotensin II (1–8) produces angiotensin III (2–8) which can be catalyzed in the biological system by aminopeptidase A, as described in Figure 1. We found that angiotensin III (2–8) potentiated the effects of angiotensin II (1–8) on spike–AXL binding (Figure 5A). Aminopeptidase N further cleaves angiotensin III (2–8) at the *N*-terminus into angiotensin IV (3–8). Angiotensin IV (3–8) further enhanced the effects of angiotensin III (2–8) on spike–AXL binding (Figure 5B). The effects of angiotensin III (2–8) and angiotensin IV (3–8) on spike–AXL binding were determined to be significantly different (Figure 5C). These results indicate that angiotensin IV (3–8) is more effective in enhancing spike–AXL binding than angiotensin III (2–8). Such a difference between the effects of angiotensin II (1–8) and angiotensin IV (3–8) was not observed in the activities of other proteins such as furin (904.0 ± 26.0 RFU for angiotensin II and 937.5 ± 41.4 RFU for angiotensin IV).

### 2.4. Effects of N-Terminal Deletion of Angiotensin (1–7) on Spike–AXL Binding

**Figure 6:**
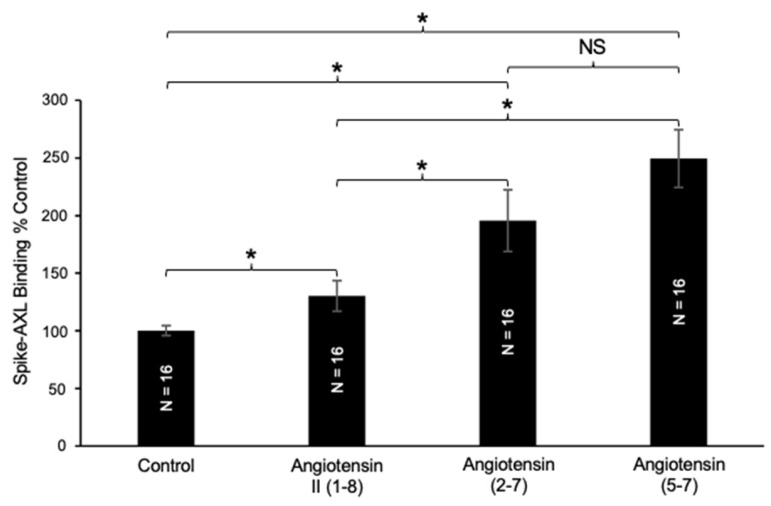
Effects of angiotensin (2–7) and angiotensin (5–7) on spike–AXL binding. Angiotensin II (1–8), angiotensin (2–7), and angiotensin (5–7) all significantly enhance the spike–AXL binding. The enhancing effects of angiotensin (2–7) and angiotensin (5–7) are more potent than those of angiotensin II (1–8), and the effects of angiotensin (2–7) and angiotensin (5–7) are comparable to each other. The bar graphs represent means ± SEM. An asterisk (*) denotes that the values are significantly different from each other at *p* < 0.05, and “NS” indicates “not significant”.

As shown in Figure 4, the effects of angiotensin II (1–8) and angiotensin (1–7) on spike–AXL binding are comparable. The *N*-terminal deletion of angiotensin (1–7) into angiotensin (2–7) further increased its ability to enhance the spike–AXL binding (Figure 6). These results suggest that the removal of Asp1 and Phe8 increases the actions of angiotensin II (1–8) on spike–AXL binding. Similarly, angiotensin (5–7) had an enhancing effect on spike–AXL binding (Figure 6), indicating that the removal of Asp1, Arg2, Val3, Tyr4, and Phe8 does not lessen the enhancing ability of angiotensin II (1–8). The difference between the effects of angiotensin (2–7) and angiotensin (5–7) was not significantly different.

### 2.5. Effects of Angiotensin II (1–8) Mutants on Spike–AXL Binding

To obtain further information on the structure–function relationship of the effects of angiotensin peptides on spike–AXL binding, we utilized commercially available angiotensin II (1–8) analogs with different amino acid alterations.

**Figure 7:**
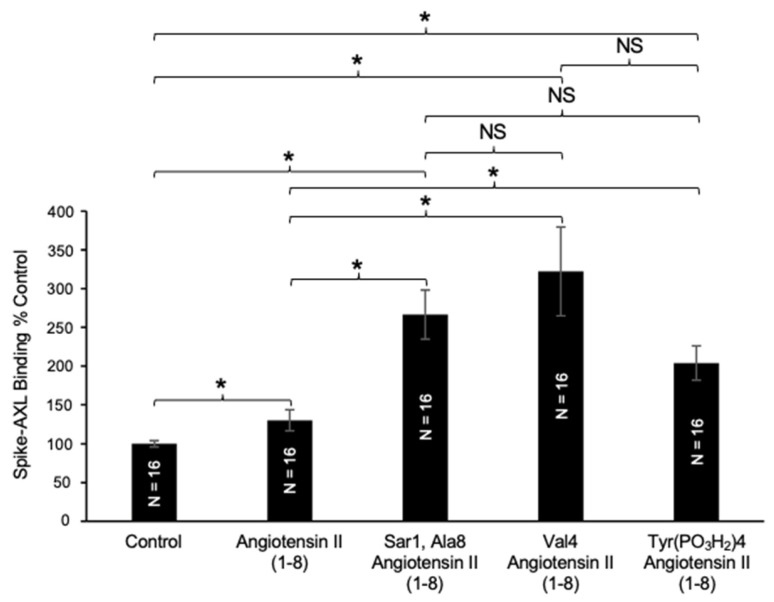
Effects of angiotensin II (1–8) analogs on Spike–AXL binding. Angiotensin II (1–8) and its analogs, Sar1, Ala8 angiotensin II (1–8), Val4 angiotensin II (1–8), and Tyr(PO_3_H_2_)4 angiotensin II (1–8), all enhance the spike–AXL binding. The enhancing effects of the analogs on spike–AXL binding were significantly greater than those of angiotensin II (1–8), but the differences among the effects of the analogs were not significant. The bar graphs represent means ± SEM. An asterisk (*) denotes that the values are significantly different from each other at *p* < 0.05, and “NS” indicates “not significant”.

The Sar1, Ala8 angiotensin II (1–8) analog replaces the Asp1 of angiotensin II (1–8) with sarcosine (*N*-methylglycine) and Phe8 with alanine. The addition of this analog increased the spike–AXL binding and the enhancing effects of this analog were significantly more effective than those of wild-type angiotensin II (1–8) (Figure 7). Despite altering the first and last amino acids of angiotensin II (1–8), this mutant still exhibited an enhancing effect on spike–AXL, suggesting that changing Asp1 and Phe8 does not remove angiotensin II (1–8)’s enhancing effect on spike–AXL binding and, instead, increases it. This is consistent with the observations that angiotensin (2–7) further potentiated the enhancing effect of angiotensin II (1–8) on spike–AXL binding when Asp1 and Phe8 are removed (Figure 6).

Next, we investigated the effects of another available angiotensin II analog, namely Val4 angiotensin II (1–8), which replaces the Tyr4 of angiotensin II (1–8) with valine. This Tyr4Val angiotensin II (1–8) mutant substantially potentiated the enhancing effects of angiotensin II (1–8) on spike–AXL binding (Figure 7). Another available angiotensin II (1–8) analog that has a modification in the tyrosine residue at position 4 is Tyr(PO_3_H_2_)4 angiotensin II (1–8), which has a phosphorylated Tyr4. Similarly, applying this analog yielded an increase in the enhancing effects of angiotensin II (1–8) on spike–AXL binding (Figure 7). The enhancing effects of the Val4 angiotensin II (1–8) analog and the Tyr (PO_3_H_2_)4 angiotensin II (1–8) analog demonstrated that modifications to the tyrosine residue at position 4 in angiotensin II (1–8) trigger the enhancement of spike–AXL binding. While these three angiotensin II (1–8) analogs all potentiated the enhancing effects of angiotensin II (1–8) on spike–AXL binding, we did not note significant differences among the effects of the three molecules (Figure 7).

### 2.6. Effects of Angiotensin IV (3–8) on Spike Protein Binding to AXL, ACE2, and NRP1

**Figure 8:**
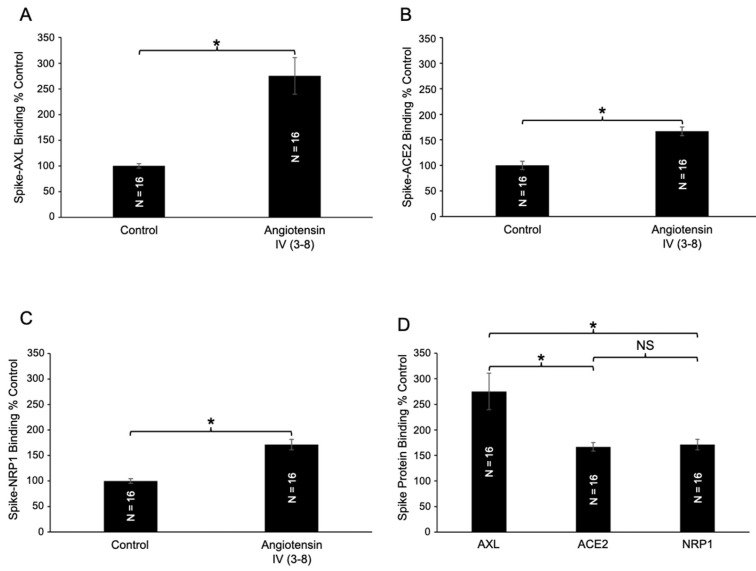
Effects of angiotensin IV (3–8) on spike–AXL binding to host cell receptors. (**A**) Angiotensin IV (3–8) significantly enhanced spike–AXL binding. (**B**) Angiotensin IV (3–8) significantly enhanced spike–ACE2 binding. (**C**) Angiotensin IV (3–8) significantly enhanced spike–NRP1 binding. (**D**) Although angiotensin IV (3–8) enhanced spike–AXL binding, spike–ACE2 binding, and spike–NRP1 binding, this peptide most potently enhanced spike–AXL binding. The bar graphs represent means ± SEM. An asterisk (*) denotes that the values are significantly different from each other at *p* < 0.05, and “NS” indicates “not significant”.

Given the impressive enhancing effect of angiotensin IV (3–8) on spike–AXL binding, we further tested its impact on other host cell receptors. In addition to significantly enhancing spike–AXL binding (Figure 8A), angiotensin IV (3–8) also increased binding of the spike protein to ACE2 (Figure 8B) and NRP1 (Figure 8C). The effects of angiotensin IV (3–8) on spike–AXL versus spike–ACE2 and spike–NRP1 were significantly different (Figure 8D), while no significant difference was observed between the spike–ACE2 and spike–NRP1 interactions. These findings demonstrate that angiotensin IV (3–8) enhances spike protein binding to AXL, ACE2, and NRP1 host cell receptors, with the most potent effects occurring on spike–AXL binding.

## 3. Discussion

SARS-CoV-2, the causative virus of COVID-19 and long COVID, utilizes its spike protein to bind host cell receptors, initiating viral entry [1,2]. In addition to the spike protein embedded in the viral particles, freely circulating spike proteins, particularly the S1 subunit proteins, can bind to these receptors and possibly exert adverse effects associated with COVID-19 complications [21,22]. The binding of the spike protein to its major host cell receptor, ACE2, results in the internalization and downregulation of ACE2 on the plasma membrane [23,24,25,26]. Since ACE2 physiologically degrades angiotensin II (1–8), its loss leads to elevated angiotensin II (1–8) levels in the circulation, a phenomenon observed in COVID-19 patients [27,28,29]. Elevated angiotensin II (1–8) levels can stimulate AT1R, promoting cardiovascular, renal, and neurological complications in COVID-19 patients [22].

**Figure 9:**
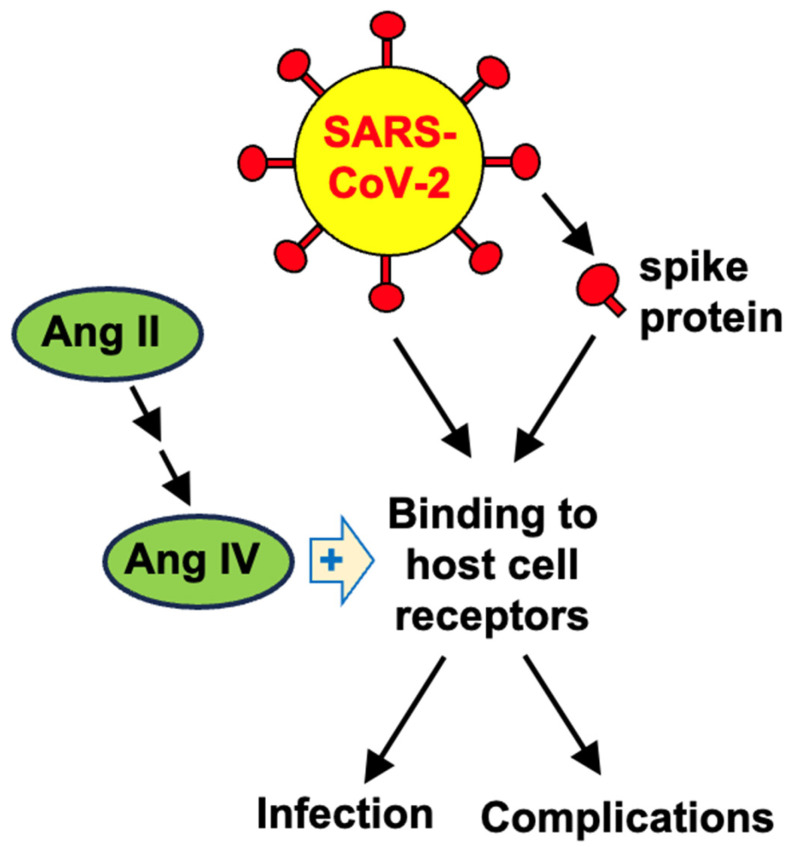
A scheme summarizing the key results of the present study.

Given the low expression levels of ACE2 in respiratory tissues [12,13], the COVID-19 pathogenesis is likely also mediated by other spike protein receptors such as AXL [14]. A key finding of the present study is that increased angiotensin II (1–8) also enhances the spike–AXL host cell receptor binding, suggesting a novel mechanism by which elevated angiotensin II (1–8) could amplify viral entry for infection and/or spike protein-mediated pathogenic effects that could lead to COVID-19-associated complications [22] (Figure 9). Another major finding is that angiotensin IV (3–8), which can be produced from angiotensin II (1–8) in the biological system, most potently enhances spike protein binding to AXL.

Caputo et al. [30] reported that angiotensin II (1–8) enhanced the spike protein-dependent infection of wild-type SARS-CoV-2 as well as pseudo-typed vesicular stomatitis virus expressing spike protein in Calu-3 human airway epithelial cells. In this study, the authors concluded that the mechanism of this event is through angiotensin II (1–8) via AT1R, increasing ACE2 mRNA and protein expression [30]. Our results from the present study point to the possibility that angiotensin II (1–8) may activate spike protein–AXL binding that, in turn, increases SARS-CoV-2 infection. Alternatively, angiotensin IV (3–8) was produced from the added angiotensin II (1–8) in the culture medium, which then enhanced spike protein binding to host cell receptors.

While the direct actions of angiotensin II (1–8) have been well studied in many pathophysiological events, especially through its receptors [3,4,5,6,7], it is not well-known that angiotensin II (1–8) can be cleaved to become other shorter peptides, which can exert other biologic events as consequences of RAS activation. As shown in Figure 1, these shorter peptides, which include angiotensin III (2–8) and angiotensin IV (3–8), are generated through the actions of aminopeptidases [3,4,5,7]. Recently, because of COVID-19, it has become well-recognized that the major spike protein target, ACE2, catalyzes the cleavage of angiotensin II (1–8) into angiotensin (1–7) [3,4]. Thus, angiotensin II (1–8) actions may not be limited to its direct effects on angiotensin receptors but may be mediated through the cleaved peptides. This raises the question of whether therapeutic means to inhibit angiotensin receptor actions would be optimal to cope with RAS-mediated pathologies.

Our structure–function study suggests that the *C*-terminal residues His9 and Leu10 in longer peptides such as angiotensin I (1–10) may interfere with the spike–AXL binding-enhancing activity, as observed in our experiments. The deletion of the *C*-terminal amino acid from angiotensin II (1–8) did not alter the peptide’s ability to enhance spike–AXL binding. In contrast, the deletion of the *N*-terminal amino acid further increased spike–AXL binding, suggesting that removing Asp1 relieved the structural interference that hinders the activation of spike–AXL binding. This could be attributed to the negative charged properties of aspartic acid. Experiments using angiotensin peptides with Asp1 replaced with glutamic acid may clarify this mechanism. Our experiments using angiotensin II (1–8) analogs indicated that modifications to tyrosine at position 4 trigger the enhancement of spike–AXL binding. We plan to conduct experiments by replacing this tyrosine with other residues, deleting this tyrosine, or making other modifications to the tyrosine side chain to further elucidate the molecular mechanisms behind these events.

In addition to the mechanism where the spike protein binding to ACE2 and subsequent downregulation of plasma membrane ACE2 is the major factor that increases angiotensin II (1–8) [22], the enhancement of spike protein binding to host cell receptors by angiotensin II (1–8) and shorter angiotensin peptides should play a critical role by amplifying the spike protein–angiotensin II cycle in COVID-19 pathology. It may also point to an important concept that individuals with hypertension and other clinical conditions, who already have higher angiotensin II (1–8) levels, may be more susceptible to SARS-CoV-2 infection, present with more severe symptoms, and be at risk of experiencing further complications.

In summary, it was determined that angiotensin II (1–8) had an enhancing effect on spike–AXL binding. A longer angiotensin peptide, angiotensin I (1–10), did not have a significant effect on spike–AXL binding. In contrast, shorter lengths of angiotensin peptides, particularly angiotensin IV (3–8), notably increased spike protein binding to AXL as well as to ACE2 and NRP1. From these results, we propose that angiotensin II (1–8), through the production of angiotensin IV (3–8), enhances spike protein binding to the host cell receptors, resulting in the exacerbation of SARS-CoV-2 infection as well as spike protein-mediated complications associated with COVID-19 (Figure 9). Currently, we do not know whether angiotensin peptides interact with the spike protein or AXL to cause the binding enhancement. Further studies to determine the molecular mechanisms of the enhancement of spike binding by angiotensin peptides as well as those extending biochemical studies in cells and in in vivo models are needed to determine the roles of angiotensin peptides in enhancing SARS-CoV-2 infection, as well as the actions of freely circulating S1 spike protein, which perhaps promotes COVID-19 complications and the symptoms that patients with long COVID are currently experiencing. These investigations should contribute to providing new therapeutic agents.

## 4. Materials and Methods

### 4.1. Chemicals

Angiotensin I (1–10), angiotensin II (1–8), angiotensin (1–7), angiotensin (1–6), angiotensin III (2–8), angiotensin (2–7), and angiotensin (5–7) were purchased from APExBIO Technology LLC (Houston, TX, USA). Angiotensin IV (3–8) was purchased from Bachem Americas, Inc. (Torrance, CA, USA). Sar1, Ala8 angiotensin II (1–8), Val4 angiotensin II (1–8), and Tyr(PO_3_H_2_)4 angiotensin II (1–8) were purchased from MilliporeSigma (Burlington, MA, USA). All angiotensin peptides were used at a final concentration of 40 μg/mL in the experiments.

### 4.2. Spike Protein Binding Assays

**Figure 10:**
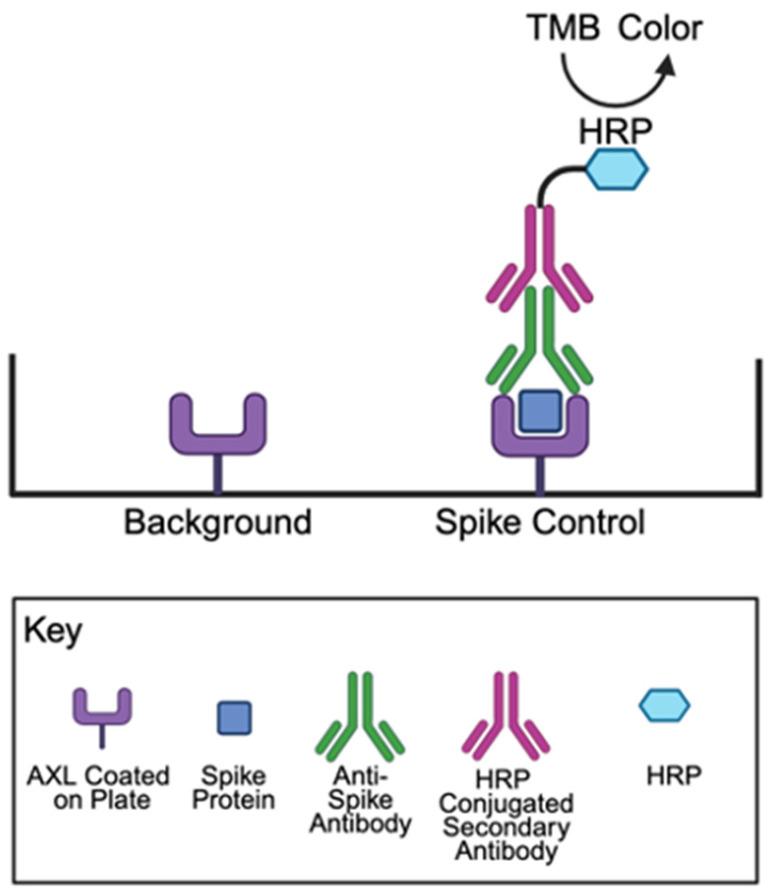
Principles of the spike protein–AXL binding assay. The RayBio COVID-19 Spike-AXL Binding Assay Kit contains AXL protein bound to the bottom of the microplate wells. SARS-CoV-2 spike protein S1 is added, washed, and the bound spike protein S1 to AXL is detected using anti-spike protein S1 IgG antibody and HRP-conjugated anti-IgG. The addition of the TMB substrate results in HRP catalyzing the reaction to produce a detectable color using an absorbance microplate reader. Created in BioRender. Oliveira, K. (2025) https://BioRender.com/s0zewuu.

A COVID-19 Spike-AXL Binding Assay Kit (Catalog #: CoV-AXLS1-1) and COVID-19 Spike-NRP1 Binding Assay Kit (Catalog #: CoV-NRP1S1-1) were purchased from RayBiotech Life, Inc. (Peachtree Corners, GA, USA). A CoviDrop SARS-CoV-2 Spike-ACE2 Binding Inhibitor Screening Fast Kit (Catalog #: D-1004-96) was purchased from Epigentek Group Inc. (Farmingdale, NY, USA). Assays were performed according to the manufacturers’ instructions, and the horseradish peroxidase (HRP) activity was measured with the 3,3′,5,5′-tetramethylbenzidine (TMB) substrate. Figure 10 depicts the principles of the spike protein–AXL binding assay. Each experiment included a negative control (buffer only), a positive control (containing angiotensin II), and test molecule groups. The reagents were added to AXL-coated microplate wells in the following order: buffer, angiotensin peptides, and the spike protein. The plate was then incubated overnight at 4 °C with shaking. After incubation, wells were washed and incubated with the primary antibody for 1 h at room temperature. This was followed by another wash and a 1 h incubation with an HRP-conjugated secondary antibody at room temperature. Following the final wash, TMB substrate solution was added and allowed to react for 30 min at room temperature. The enzymatic reaction was then stopped, and the absorbance was measured at 450 nm using an EMax Plus Microplate Reader (Molecular Devices, LLC., San Jose, CA, USA).

### 4.3. Furin Activity Assay

The Fluorimetric SensoLyte Rh110 Furin Activity Assay Kit (AnaSpec Inc., Fremont, CA, USA; Catalog #: AS-72256) was used to monitor the furin protease activity. The cleavage of the fluorogenic substrate by furin generates the rhodamine 110 fluorophore, which was measured using a POLARstar OPTIMA Fluorescence Microplate Reader (BMG Labtech, Cary, NC, USA). Results at the 120 min time point are reported in relative fluorescence units (RFU).

### 4.4. Statistical Analysis

All data are expressed as mean ± standard error of the mean (SEM). The control groups without angiotensin and the experimental groups with angiotensin were compared using unpaired *t*-tests. Statistical significance was defined at *p* < 0.05.

## Data Availability

Data are available upon request.

## Abbreviations

The following abbreviations are used in this manuscript:

SARS-CoV-2	Severe acute respiratory syndrome coronavirus 2
COVID-19	Coronavirus disease 2019
ACE2	Angiotensin-converting enzyme 2
RAS	Renin–angiotensin system
ACE	Angiotensin-converting enzyme
AT1R	Type 1 angiotensin II receptor
GPCR	G protein-coupled receptor
ADH	Antidiuretic hormone
NO	Nitric oxide
AT2R	Type 2 angiotensin II receptor
HRP	Horseradish peroxidase
TMB	3,3′,5,5′-tetramethylbenzidine
RFU	Relative fluorescence unit
SEM	Standard error of the mean
